# Assessment of the olive oil extraction plant layout implementing a high-power ultrasound machine

**DOI:** 10.1016/j.ultsonch.2021.105505

**Published:** 2021-03-04

**Authors:** Antonia Tamborrino, Agnese Taticchi, Roberto Romaniello, Claudio Perone, Sonia Esposto, Alessandro Leone, Maurizio Servili

**Affiliations:** a Department of Agricultural and Environmental Science, University of Bari Aldo Moro, Via Amendola 165/A, 70126 Bari, Italy; b Department of the Science of Agriculture, Food and Environment, University of Perugia via S. Costanzo, 06126 Perugia, Italy; c Department of Agriculture, Food, Natural Resources and Engineering (DAFNE), University of Foggia, Via Napoli, 25 – 71122 Foggia, Italy

**Keywords:** High-power ultrasound, Malaxation, Viscosity, Olive oil quality, Extractability, Rheology

## Abstract

The objective of this study is to assess the effects of installation and operation of a high-power ultrasound machine (HPU) for the treatment of olive paste by using ultrasound technology in order to evaluate the best way installation and the best definition of the operating conditions of the machine. The study was conducted installing in an industrial olive oil mill a continuous processing ultrasound machine, which used a frequency of 20 kHz able to work at 3200 kg h^−1^ as feed capacity. Checking of performance has been carried out by the assessment of the different operating and process conditions, assessing in particular the impact of the ultrasound treatment before and after the malaxation phase on performance indicators of the continuous olive oil plant (plant extractability, olive paste rheological characteristic) and on selected chemical properties of the olive oil extracted (quality parameters, antioxidant content, and volatile profiles). In the tested conditions, high-power ultrasound treatment did not produce significant effect on the legal parameters (free acidity, peroxide index and spectrophotometric indexes), while a significant increase in the content of phenolic compounds was generally observed; higher enhancements were more evident when the high-power ultrasound treatment was carried out before the malaxation phase.

## Highlights

• A high power ultrasound machine was implemented in an industrial olive oil mill.
• Two different arrangements of installation were tested.
• Best quantitative results obtained when the HPU was between crusher and malaxer.
• No modifications in qualitative parameter occurred by using the HPU.
• Extractability value raised when HPU was used.

## 1 Introduction

Recently, several industrial and academic investigations are conducting on the development of innovative virgin olive oil (VOO) extraction processes. They focused on the treatment of the olive paste using heat exchangers, working at a temperature below 27–28 °C [1], [2], [3], [4] and by mild physical technologies, such as microwave-assisted systems [5], [6], low-frequency ultrasound [7], [8], [9], [10], [11], [12], [13], high-frequency ultrasound [14], [15], [16], [17], and pulsed electric field [18], [19]. These technologies aimed to increase the plants working capacity, therefore, to achieve a positive impact on the extractability of the decanter and, at the same time, on the continuity of the process and, in several cases, on olive oil quality. Of these, ultrasound extraction seems to have several advantages due to its mechanical and thermal effects, which do not increase energy and water needs compared to the conventional processes [10], [20], [21], [22]. Mason [23] has defined the ultrasounds as sound wave frequencies, not audible for the human ear, from 20 Hz up to 20 kHz. These ultrasonic waves are generally classified as high-power ultrasounds (HPU) (20 kHz) and diagnostic ultrasounds (higher than 1 MHz). The physical effect of HPU application on olive paste creates a mechanical movement generated by the high and low-pressure cycles. The resulting mechanical movement with the shear forces leads to the increment of mass transfer and also the rupture of cell walls [24], [25]. In the last decades, the application of HPU was carried out by laboratory scale and by industrial scale plant. One of the first application in olive oil extraction process of high-power low frequency ultrasound before the malaxing of olive paste was carried by Jimenez et al. [20] by a direct and indirect application. The high-power ultrasound olive paste treatment improved the oil extractability without any changes of the virgin olive oil quality parameters (free acidity value, peroxide value, K270, and K232), whereas the sonicated olive paste gave a VOO with lower bitterness and higher content of tocopherols, chlorophylls, and carotenoids. Related to sensory characteristics, off-flavour volatiles were not detected in oils from sonication treatments, showing the higher intensity of positive organoleptic attributes than those untreated [22].

Later Bejaoui et al. [10] proposed a laboratory-scale device for HPU pre-treatment of olive paste. The sonication treatment resulting in an improvement of the oil extractability by 5.74% compared to conventional malaxation and no alteration in the quality indices, fatty acid composition, and volatile aromatic compounds of the VOO. Besides, the fat autoxidation mechanism was not accelerated by this treatment. Meanwhile, the VOO obtained from olive pastes treated with high-power ultrasound treatment showed higher content of tocopherol, chlorophylls, and carotenoids, while a reduction in phenolic content and bitterness index was observed too.

Additionally, Bejaoui et al. [11], [26] tested the effect of 3 different ultrasounds frequencies at a semi-industrial scale showing an increase of yield and VOO characteristics. Furthermore, the multi-frequency HPU technique tested as aid or substitute for the olive paste malaxation step. The first effect observed for all the frequencies application on the olive paste when compared with the untreated paste was the rapid heating. Regarding the process efficiency, in general, the application of high-power ultrasound technology improves the process yield compared to the untreated paste. Concerning the VOO quality and nutritional compounds, no alteration was shown on the volatile compounds, related to the positive sensorial attribute, showed similar levels to those from conventional treatment, and those related to the off-flavors were not observed. Therefore, the high-power ultrasound treatment gave Extra VOO with a more equilibrated sensorial profile. Later the positive effect on the laboratory scale investigation, different researches have been conducted on industrial scale up [5], [12], [27] have confirmed the positive effects of the sonification with low frequency ultrasound. Besides, Servili et al. [12] employed the high-power ultrasound technology testing its action on the extraction of olive oil at the industrial scale using different pressure level (1.7 bar and 3.5 bar) generated in the US cell. Increases of extractability were registered when olive paste was treated at 3.5 bar. However, it was investigated the influence of high frequency ultrasound in the range 300–800 kHz on olive oil extractability [17], [28], [29], [30]. This employment of frequency requires the application of specific reactors and the treatment required after malaxation and before centrifugation due to the specific separation intervention attributed to these ultrasound frequencies [8], [14], [29], [30], [31]. These studies demonstrate the enhancement of olive oil separation, thereby increasing the extraction yield.

The high-power ultrasound at present time have been tested by implementing this technology between the olive crushing phase and the conditioning phase of the olive paste by laboratory and industrial-scale apparatus. However, also to facilitate operative installation in oil mills, it could be useful to evaluate the performance of industrial olive oil extraction plant when a high-power ultrasound (HPU) machine is assembled between the conditioning phase of the olive paste and the solid-liquid separation phase. In this scientific paper the checking of performance of the HPU machine has been carried out by the assessment of the impact of the high-power ultrasound treatment before and after the conditioning phase of the olive paste.

## 2 Materials and methods

### 2.1 Industrial olive oil extraction plant and high-power ultrasound (HPU) machine

Comparative tests to evaluate the effect of HPU machine, implemented before and after the conditioning phase, versus quantitative parameters of the plant and qualitative parameters of the olive oil were performed in an industrial scale olive oil mill (Oleificio Cericola s.r.L.s., Foggia-Italy). The industrial olive oil extraction plant was constituted by a defoliator, a washing machine (Special Automatic, Alfa Laval Corporate AB, Lund, Sweden), a hammer crusher combined with a partial de-stoner (Pietro Leone e Figli s.n.c., Foggia, Italy), an olive pastes conditioning unit made of a mixing-coil heat exchanger, as reported in Tamborrino et al. [5], combined with a 6 malaxers sealed on the top of 700 L capacity each, a three-phase solid-liquid decanter centrifuge (NX X32, Alfa Laval Corporate AB, Lund, Sweden), and a liquid-liquid vertical centrifuge (UVPX 507, Alfa Laval Corporate AB, Lund, Sweden).

HPU machine involved a continuous treatment of the olive pastes with low frequency – high-power intensity ultrasound. The ultrasonic equipment (Hielscher Gmbh, Teltow, Germany), installed by Seneco Science (Seneco s.r.L., Milano, Italy), consists of an electrical power generator, which provides the source of energy to drive the transducer; a transducer, an electromechanical part of the processor to convert the electric power into mechanical oscillations and generate the actual ultrasound; a booster, to increase or reduce the mechanical amplitude of the emitter; and an emitter, which consists of a sonotrode that physically sends the ultrasonic waves into the medium to be sonified (i.e., olive paste). Table 1 shows the main characteristics of the ultrasound equipment. The ultrasonic processor creates longitudinal mechanical oscillations with a frequency of 20 kHz by electric stimulation (reversed piezoelectric effect). The power input can be selected continuously between 60% and 100% of the maximum power. The sonotrode mounted to the horn tip increases the oscillations and transfers them into the olive paste mainly via its front face. The ultrasonic oscillations on the sonotrode generate cavitation in the medium and enables the desired effects of high intensity “power” ultrasound for the extraction of oil [32]. The olive paste flows inside a stainless-steel cell in which the sonotrode is installed vertically. An automatic system made it possible to adjust the pressure of the paste inside the sonication cell based on the value previously set on the PLC.

**Table 1:** Ultrasonic equipment characteristics.

Ultrasonic processor (generator + trasducer)	UIP4000hdT	Amplitude control	60%–100%, continuous
		Max Amplitude	30 μm

Booster	B4-1.4	Max Amplitude (booster)	30 μm
		Max Amplitude (reducer)	59 μm

Sonotrode	BS4d22	Frontal Area	41 cm^2^
		Amplitude at 100% (no booster)	42 μm

The sonotrode (Table 1) provide a power intensity of about 100 W/cm^2^ (unit of sonotrode surface equal to 41 cm^2^) at operating pressure of 3.5 bar, and thus an average total power of about 4100 W. When the booster is employed the power intensity increases to 150 W/cm^2^ (at 3.5 bar), and the total power for the treatment reaches about 6150 W.

### 2.2 Experimental plant design

**Fig. 1:**
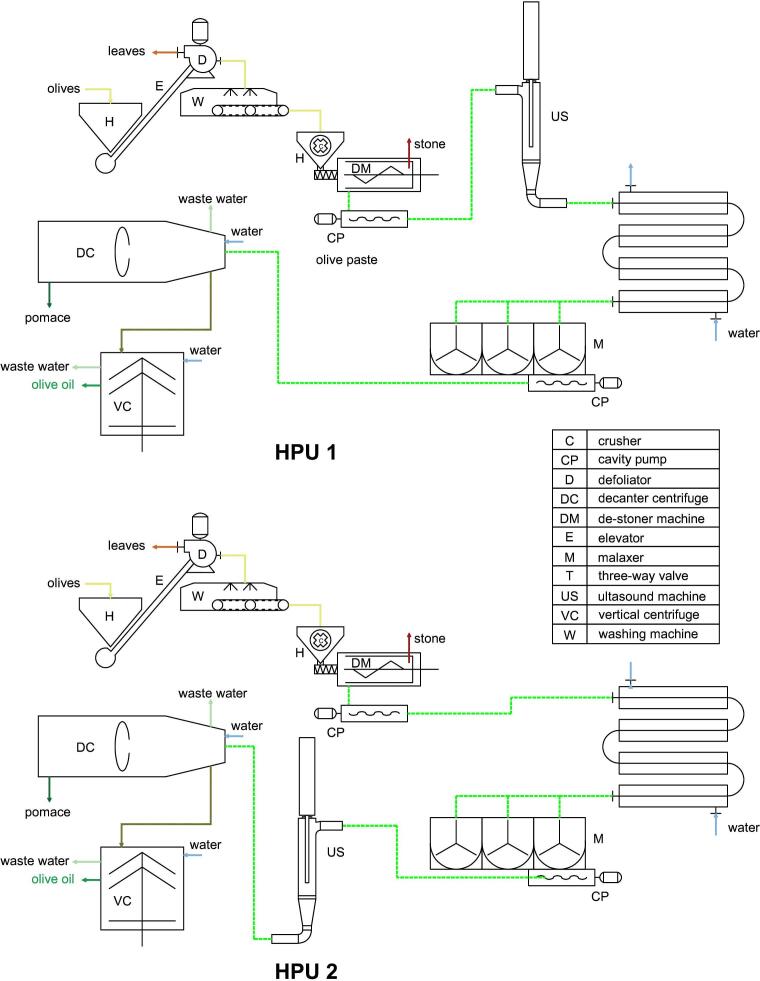
Plant layout arrangements of the olive oil extraction process line.

Fig. 1 shows the olive oil extraction plant in the two different layout arrangements with the HPU machine installed before the conditioning unit and downstream it.

Below three different plant layout configurations are reported, two configurations operate with the HPU machine and one represents the control layout configuration without HPU machine:

– Configuration 1: HPU machine positioning upstream olive paste conditioning unit (HPU 1);
– Configuration 2: HPU machine positioning downstream olive paste conditioning unit (HPU 2);
– Configuration 3: HPU machine was uninstalled (Control)

Switching for the different operating modes was manually, by removing the ultrasound unit prior the malaxing machine, and by connecting it to the exit of the latter. The performance of the plant in arrangement 1 and 2 were compared with the control thesis. During the tests, the pressure level set in the sonication cell was 3.5 bar, which is considered an optimal value, as demonstrated in Servili et al. [12].

Experimental tests were performed using olives of cultivar Peranzana (*Olea europaea* L.), mechanically harvested in Torremaggiore (Foggia) having a maturity index of 1.8 determined as reported in Uceda et al. [33] and processed within 2 h of the harvest. Each configuration was performed five times by processing homogenous olive batches (700 kg per batch). During the tests malaxation was performed for 30 min at 25 ± 1 °C, plant mass flow rate of 3200 kg h^−1^ and dilution water to the decanter of 350 L h^−1^. Five runs that each arrangement was made.

### 2.3 Characterization of olive paste

#### 2.3.1 Moisture and oil content in olives and pomace

Moisture content (% w/w) was detected by drying the milling olives or pomace sampled from the decanter at 105 °C to constant weight and the total oil content was detected following the analytical technique described by Cherubini et al. [34].

#### 2.3.2 Rheological characteristic

Three replicates of olive paste samples for each run were collected at the outlet of crusher, malaxer and ultrasound machines, for both arrangements 1 and 2. A Brookfield rotational viscometer model DV2-HBT (Brookfield Engineering Laboratories, Inc., Stoughton, MA, USA) equipped with interchangeable disc spindles, 2–7 (model RV/HA/HB; Brookfield DVII + Brookfield Engineering Laboratories) was used for rheological analysis of olive paste samples. The viscosity measurement was carried out using 600 mL of olive paste, loaded into 1000 mL glass containers conditioned at 27 °C in a thermostatic bath. The apparent viscosity of each sample was recorded at 10 rotational speeds from 0.5 to 100 rpm using the RV/HA/HB-4 spindle. To interpret the experimental results in terms of viscosity, the torque-speed data and scale readings were converted to shear stress and shear speed ratios using numerical conversion values. An empirical power-law model was used to calculate the apparent viscosity and flow behaviour index from the shear rate, using the power law equation η_app_ = Kγ^n−1^, where η_app_ is the apparent viscosity, γ is the shear rate (s^−1^), n is the flow behaviour index (without size), K is the consistency index (Pa s^n^). The result is expressed per each arrangement as the mean of fifteen data obtained from the measurements taken on the three samples of pasta per each of the five runs carried out.

### 2.4 Olive oil analysis

#### 2.4.1 Solvents and reference compounds

Methyl alcohol HPLC grade and glacial acetic acid were purchased from VWR International Srl (Milan, Italy), water HPLC grade was obtained by using purification units (ELGA, Germany). Hydroxytyrosol (3,4-DHPEA) and tyrosol (*p*-HPEA) were supplied by Cabru s.a.s. (Arcore, Milan, Italy). The dialdehydic forms of elenolic acid linked to 3,4-DHPEA and *p*-HPEA (3,4-DHPEA-EDA and *p*-HPEA-EDA, respectively), the isomer of oleuropein aglycon (3,4-DHPEA-EA), (+)-1-acetoxypinoresinol were extracted from virgin olive oil according to the procedure reported from Selvaggini et al., 2014. (+)-Pinoresinol was purchased from PhytoLab GmbH & Co. KG (Vestenbergsgreuth, Germany). Pure analytical standards of volatile compounds were obtained from Merck (Milan, Italy).

#### 2.4.2 Legal quality parameters

The legal quality parameters of the VOO samples (free acids, peroxide index, K_232_, K_270_ and ΔK) were evaluated according to the Regulation (EU) 2019/1604 (OJEC, 2019) [35].

#### 2.4.3 Phenolic compounds

The hydrophilic phenols of VOO were extracted according to Taticchi et al. (2020). The phenolic extract obtained was solubilized with 1 mL of a solution composed of methanol/water (50:50 v/v) and filtered with a 0.2 µm PVDF syringe filter (Agilent Technologies, Santa Clara, CA, USA). The qualitative and quantitative analysis of the phenolic compounds of the extract was conducted by HPLC using an Agilent Technologies system Mod. 1100 (Agilent Technologies, Santa Clara, CA, USA), composed of a vacuum degasser, a quaternary pump, an autosampler, a thermostated column compartment and diode array detector (DAD) and equipped with a C18 column (Spherisorb ODS-1 (250 mm × 4.6 mm) 5 μm particle size, supplied by Waters S.p.A. (Milan, Italy). The mobile phase was composed of 0.2% acetic acid (pH 3.1) in water (solvent A) and methanol (solvent B), and the gradient was modified as follows: 95% A/5% B for 2 min, 75% A/25% B in 8 min, 60% A/40% B in 10 min, 50% A/50% B in 16 min, 0% A/100% B in 14 min. This composition was maintained for 10 min and was then returned to the initial conditions and equilibration in 13 min. The total running time was 73 min. The phenolic compounds were detected by using the DAD set at a wavelength of 278 nm. The quantification of phenolic compounds was determined by using single calibration curves for each compound and the results are expressed as mg kg^−1^.

#### 2.4.4 Volatile compounds

The evaluation and quantification of volatile compounds in EVOOs were done by headspace-solid phase microextraction followed by gas chromatography-mass spectrometry analysis (HS-SPME-GC/MS) according to Taticchi et al. (2020), briefly 3 g of VOO were placed in a 20 mL vial and 2-methylpropyl acetate was added as an internal standard at a concentration of 9.8 mg/kg. The vial was kept for 10 min at 35 °C to allow the equilibration of the volatiles in the headspace. Then the SPME fiber (a 50/30 μm divinylbenzene/Carboxen/poly(dimethylsiloxane) (DVB/CAR/PDMS) with a length of 2 cm; StableFlex (Supelco, Inc., Bellefonte, PA, USA) was exposed to the vapor phase for 30 min for sampling the volatiles. The analyses of these compounds were performed with an Agilent Technologies GC 7890B equipped with a “Multimode Injector” (MMI) 7693A (Agilent Technologies, Santa Clara, CA, USA) and a thermostated PAL3 RSI 120 autosampler provided of a fiber conditioning module and an agitator (CTC Analytics AG, Zwingen, Switzerland). The detection system was an Agilent 5977B single quadrupole GC/MSD with an EI Extractor (XTR) source (Agilent Technologies, Santa Clara, CA. USA). The volatiles adsorbed by the fiber were thermally desorbed in the hot GC injector port, which was set in splitless mode, for 5 min at 250 °C. The volatile compounds were separated on a DB-WAXetr column (50 m, 0.32 mm i.d., 1 μm film thickness) (Agilent Technologies, Santa Clara, CA, USA) using the same conditions reported in Taticchi et al. (2020). The volatile compounds were identified by comparison of their mass spectra and retention times with those of authentic reference compounds and with the spectra in the NIST 2014 mass spectral library. For quantification, calibration curves for each compound by internal standard calculation were constructed and the results are expressed as µg kg^−1^.

### 2.5 Statistical analysis

The machine learning and statistic toolbox of MATLAB® was used to process the experimental data. The significance among means of group of data was detected by the one-tailed *t*-test hypothesis test (p < 0.05).

## 3 Results and discussion

### 3.1 Effects of the ultrasound treatment and the HPU machine installation position on the rheological characteristic of the olive paste

The viscosities of non-Newtonian fluids are usually modelled by a nonlinear function of fluid strain rate and rheological parameters [36]. The consistency index K measures the average viscosity of the non-Newtonian fluid, and “n” is the flow behaviour index that measures the deviation of the fluid from Newtonian flow. It is the shear-thickening fluid when n > 1, and the shear-thinning fluid when n < 1. The rheological parameters dominate the trend and extent of the effect of the change in strain rate on the viscosity, in particular the consistency index is an important factor from a technological point of view useful for describing important rheological changes of a matrix.

In the present study the collected data were processed through linear regression in a bi-logarithmic scale by using the power law model, as reported in Ref. [37]. The shear rate was determined as a function of the torque and rotational speed of viscometer and used to estimate consistency and flow index. These two parameters allow calculating the apparent viscosity for each rotational speed. The difference between the actual and estimate values give a good insight on the reliability of the model. Table 2 shows the mean and standard deviation values of the estimate consistency (K) and flow behaviour index (n) for each treatment in both arrangements. In addition, the mean and standard deviation values of the coefficient of determination (R^2^) are also reported. The high R^2^ obtained in each test demonstrates that the linear regression by means of the power model properly fits the experimental data.

**Table 2:** Consistency and flow behaviour index for olive paste obtained by the plant layout arrangements.

Plant layout arrangement	After Crusher Machine			After Hpu Machine			After Malaxer Machine		
	*K*	*n*	*R^2^*	*K*	*n*	*R^2^*	*K*	*n*	*R^2^*
HPU 1	143.812 ± 7.702 *aA*	0.313 ± 0.021 *aB*	0.999 ± 0.001 *aA*	134.06 ± 13.098 *a*	0.319 ± 0.016 *a*	0.999 ± 0.000 *a*	85.627 ± 11.940 BB*	0.393 ± 0.022 *A*	0.99 ± 00.990 *B*

	After Crusher Machine			After Malaxer Machine			After Hpu Machine		
	*K*	*n*	*R^2^*	*K*	*n*	*R^2^*	*K*	*n*	*R^2^*

HPU 2	164.684 ± 8.675 *aA*	0.305 ± 0.030 *aA*	0.999 ± 0.000 *aA*	122.379 ± 7.258 *b*	0.285 ± 0.042 *a*	0.994 ± 0.004 *a*	120.443 ± 16.797 BA*	0.282 ± 0.043 *A*	0.994 ± 0.002 *B*

Data are presented as mean and standard deviation of a group of 15 data per each group. Lower case letters refer to the comparison between crusher and the first next machine in the extraction process (HPU machine in arrangement 1 and malaxer in arrangement 2); Capital letters refer to di comparison between crusher and the second next machine in the extraction process (malaxer in arrangement 1 and HPU machine in arrangement 2). Different letters in rows indicate statistically significant difference among means (*t*-test, p < 0.05). Capital letters with asterisk refer to the comparison between the last machines of the two arrangements (malaxer and HPU machine respectively). Different asterisk letters in column denote statistically significant difference among means (*t*-test, p < 0.05).

In the configuration 1 the action of the HPU machine produced no significant difference in the consistency and flow behaviour index. However, K values remarkably decreased in olive paste after the subsequent malaxation. In the arrangement 2 the malaxer machine reduced the consistency index of the crushed paste, while the following sonication treatment had no significant effect. However, it is worth to note that the influence of HPU machine on the olive paste after crushing (HPU 1) allowed to obtain a final consistency index K (after malaxation) significantly lower than that of obtained in HPU 2 (asterisked letters in Table 2). This means that the sonication of the olive paste immediately after crushing effectively reduces the viscosity of the matrix during the next malaxation. On the contrary, in HPU 2, the HPU machine treatment had no influence on the rheology of the matrix after malaxation. The obtained results suggest that the olive paste from the HPU 2 were less susceptible to the effect of deformation during shearing (at lower shear stress values applied). This indicates a less viscous behaviour of the paste conditioned according to the arrangement 1. As a result, the final consistency index of the olive paste in arrangement 1 (HPU 1) is significantly lower that arrangement 2 (HPU 2) (Table 2). Accordingly, the HPU machine installed before the malaxer machine seems to be the best choice to reduce the consistency of the olive paste and enhance the extraction yield, as reported in Section 3.2.

The results of this study clearly demonstrated that the high-power ultrasound treatment is involved in the generation of physicochemical changes in the olive paste matrix. This treatment are mainly implicate on the cavitation process due to ultrasound waves that causes disruptive effects on the fruit cell [14], and this effect appears to be more effective if it occurs in the matrix immediately after the rupture of the cells which occurs with crushing.

### 3.2 Effects of the ultrasound treatment and the HPU machine installation position on the efficiency of the plant

The olive oil extraction plant equipped by HPU machine shown different behaviour in terms of quantitative performance when the HPU machine was operative or not as reported in Table 3. First of all, the position of the machine in the chain played an important role to define its best performances. Best results in terms of quantitative performance were obtained when the HPU machine was placed between crusher and malaxer machine (HPU 1). The extractability value resulted significantly higher than that obtained in the Control. This result is confirmed by the data of the oil contained in the pomace equal to 14.92 (% dry basis) for the control and 11.54 (% dry basis) for the HPU 1 arrangement. Considering the HPU 2 arrangement by placing the HPU machine between the malaxer and decanter, the extractability value resulted significantly higher also than the control, but lower than that obtained using the HPU 1 arrangement. For this last comparison, no significant difference was highlighted for oil content in the pomace.

**Table 3:** Quantitative data for the plant layout arrangements.

Plant layout arrangements	Pomace composition		Extractability(%)
	Oil (% wet basis)	Oil (% dry basis)	
CTRL	5.70 ± 0.31 *aA*	14.92 ± 0.75 *aA*	75.46 ± 0.92 *bB*
HPU 1	4.33 ± 0.70 *b*	11.54 ± 1.35 *b*	81.40 ± 1.31 *a*
HPU 2	5.60 ± 0.60 *A*	14.58 ± 1.72 *A*	78.03 ± 1.62 *A*

Different letters in columns denote significant statistical differences at p < 0.05 (one-tailed *t*-test). Lowercase letters refer to CTRL-HPU 1 comparison. Capital letters refer to CTRL-HPU 2 comparison.

The present research follows the trend of other ones conducted in the past. In fact, Bejaoui et al. [26], in the 2017, founded a significant yield increase when high-power ultrasounds were applied to the olive paste before the centrifugation; the study was conducted at semi-industrial scale. In the same year, Leone et al. [29] tested at industrial scale a high frequency ultrasound machine placed before the horizontal centrifuge. In this case, an increase of about 2% of extractability was observed, in comparison with the control test, working with olives of Coratina variety. In 2019, same results were obtained by Servili et al. [12] where using a low frequency ultrasound machine placed before the malaxer machine an increase of extractability of 4.4% and 4.6% was obtained respectively using olives of Nocellara and Coratina cultivars.

### 3.3 Effects of the ultrasound treatment and the HPU machine installation position on the olive oil quality

The application of *ultrasound* in both arrangements during the mechanical oil extraction process showed no significant differences regarding the analysis of free acidity, peroxide index, K_232_, K_270_ and ΔK in VOOs respect to the Control trials (Table 4).

**Table 4:** Standard virgin olive oil parameters specified by the Reg (EU) 2019/1604.*

Parameters	Legal limits for EVOO	Plant layout arrangements		
		CTRL	HPU 1	HPU 2
Free acidity (%)	≤0.80	0.40 ± 0.01 *aA*	0.41 ± 0.03 *a*	0.43 ± 0.05 *A*
Peroxide index (meq O_2_ kg^−1^)	≤20.0	5.8 ± 0.4 *aA*	5.9 ± 0.5 *a*	6.1 ± 0.3 *A*
K232	≤2.50	1.789 ± 0.11 *aA*	1.750 ± 0.06 *a*	1.776 ± 0.06 *A*
K270	≤0.22	0.163 ± 0.02 *aA*	0.152 ± 0.01 *a*	0.155 ± 0.01 *A*
ΔK	≤0.01	0.002 ± 0.0005 *aA*	0.001 ± 0.0004 *a*	0.002 ± 0.0003 *A*

* Different letters in rows denote significant statistical differences at p < 0.05 (one-tailed *t*-test). Lowercase letters refer to CTRL-HPU 1 comparison. Capital letters refer to CTRL-HPU 2 comparison.

The olive oil extraction plant equipped by HPU machine produces a significant increase in the release of phenolic compounds into the oily phase compared to the corresponding control oil (Table 5). This increase mainly affected the most abundant oleuropein derivative, the dialdehydic forms of elenolic acid linked to 3,4-DHPEA (3,4-DHPEA-EDA) while the ligstroside derivatives and lignans have been no significant changes, as reported in other studies on the technological innovation of olive oil mechanical extraction process [12], [27]. The highest percentage increase of phenols (40.6%) was obtained when HPU machine was placed between crusher and malaxer machine (HPU 1), instead when the second configuration was used, more contained increases were observed (24.4%). This could be due to a more intense release of intracellular contents into the liquid medium before the malaxation phase when the olive tissues, cell walls and membranes are not completely disrupted. Moreover, great part of the coalescence process and solubilization phenomena of phenolic compounds into the oily phase are finished at the end of malaxation step, reducing the improving effect of the ultrasound treatment.

**Table 5:** Phenolic composition of EVOOs. Data expressed as mg kg^−1^.*

Phenolic compounds	Plant layout arrangements		
	CTRL	HPU 1	HPU 2
3,4-DHPEA	1.2 ± 0.04 *aB*	0.5 ± 0.1*b*	2.2 ± 0.7 *A*
*p-*HPEA	2.1 ± 0.03 *aA*	1.8 ± 0.3 *a*	2.0 ± 0.1 *B*
Vannilic acid	0.3 ± 0.03 *aA*	0.3 ± 0.1 *a*	0.1 ± 0.0 *B*
3,4-DHPEA-EDA	100.4 ± 11.7 *bB*	168.5 ± 8.3 *a*	163.3 ± 2.0 *A*
*p*-HPEA-EDA	46.3 ± 2.5 *aA*	51.1 ± 4.9 *a*	37.6 ± 0.7 *B*
(+)-1-Acetoxypinoresinol	9.8 ± 0.2 *aA*	11.4 ± 2.8 *a*	9.3 ± 1.1 *A*
(+)-Pinoresinol	9.6 ± 0.3 *aA*	11.2 ± 1.9 *a*	8.1 ± 0.6 *B*
3,4-DHPEA-EA	25.7 ± 2.8 *bA*	30.6 ± 2.6 *a*	22.7 ± 3.2 *A*
Ligstroside aglycone	4.7 ± 1.1 *aA*	5.8 ± 1.0 *a*	3.8 ± 1.0 *A*
Total phenols	200.1 ± 12.7 *bB*	281.3 ± 10.9 *a*	249.0 ± 5.3 *A*

* Different letters in rows denote significant statistical differences at p < 0.05 (one-tailed *t*-test). Lowercase letters refer to CTRL-HPU 1 comparison Capital letters refers to CTRL-HPU 2 comparison.

The impact of high-power ultrasound treatment on the volatile fraction is influenced by many factors included genetic origin of the olives and the maturation index, as reported by other studies [27]. In this experimental research, the different classes of volatile compounds (aldehydes, alcohols, esters and ketones) showed a slight reduction of their content in the VOOs extracted with ultrasound before and after the malaxation phase with the only exception of aldehydes of the HPU1 sample (Table 6). The lipoxygenase pathway (LOX), which is activated during the crushing phase, is probably stimulated by the high mass transfer induced by HPU on crushed olive paste promoting an improvement of hydroperoxide lyases (HPL) activity level. The general reduction of volatile compounds, at the end of coalescence process could be explained by a more intense stripping phenomenon directly induced by the bubble growth present in the oily phase during the acoustic cavitation process as recently showed by Gila et al. [38], analyzing the ultrasound treatments of virgin olive oil.

**Table 6:** Volatile compounds detected in EVOOs. Data expressed as μg kg^−1^.*

	Plant layout arrangements		
	CTRL	HPU 1	HPU 2
*Aldehydes*			
Pentanal	17 ± 6 *bA*	30 ± 3 *a*	16 ± 1 *A*
(*E*)-2-Pentenal	8 ± 1 *aB*	13 ± 4 *a*	9 ± 0.1 *A*
Hexanal	774 ± 82 *aA*	640 ± 67 *b*	601 ± 8 *b*
(*E*)-2-Hexenal	5597 ± 524 *bA*	6916 ± 376 *a*	5508 ± 428 *A*
(*E*,*E*)-2,4-Hexadienal	41 ± 6 *bA*	50 ± 2 a	39 ± 0.1 *A*
Ó of the aldehydes at C_5_ and at C_6_	6437 ± 531 bA	7648 ± 382 *a*	6173 ± 428 *A*

*Alcohols*			
1-Pentanol	73 ± 8 *aA*	45 ± 13 *b*	64 ± 5 *A*
1-Penten-3-ol	163 ± 13 *aA*	164 ± 15 *a*	135 ± 32 A
(*E*)-2-Penten-1-ol	26 ± 2 *aA*	19 ± 8 *a*	23 ± 7 *A*
(*Z*)-2-Penten-1-ol	127 ± 9 *aA*	113 ± 16 *a*	97 ± 12 *B*
1-Hexanol	1001 ± 6 *a*	991 ± 62 *a*	863 ± 106 *B*
(*E*)-2-Hexen-1-ol	4827 ± 27 *aA*	4058 ± 464 *b*	4381 ± 450 A
(*E*)-3-Hexen-1-ol	14 ± 1 *aA*	7 ± 4 *b*	10 ± 1 *B*
(*Z*)-3-Hexen-1-ol	397 ± 2 bA	414 ± 46 a	334 ± 44 *B*
Benzyl alcohol	36 ± 3 *aA*	36 ± 5 *a*	35 ± 8 *A*
Phenylethyl Alcohol	91 ± 10 *aA*	84 ± 13 *a*	90 ± 16 *A*
Ó sum of alcohols at C_5_ and at C_6_	*6628 ± 33 aA*	*5813 ± 471 b*	*5907 ± 466 B*

*Esters*			
Hexyl acetate	457 ± 11 *aA*	351 ± 20 *b*	355 ± 32 *B*
(*Z*)-3-Hexenyl acetate	719 ± 46 *aA*	634 ± 57 *a*	629 ± 29 *B*
Ó sum of esters at C_6_	1176 ± 47 *aA*	984 ± 60 *b*	984 ± 43 *B*

*Ketones*			
3-Pentanone	456 ± 12 *aA*	377 ± 34 *b*	372 ± 41 *B*
1-Penten-3-one	1 ± 1 *aA*	30 ± 17 b	1 ± 0 A
6-Methyl-5-hepten-2-one	28 ± 2 aA	27 ± 2 a	29 ± 1 A
Ó sum of ketones at C_5_ and at C_8_	*484 ± 12 aA*	*433 ± 38 b*	*402 ± 41 B*

* Different letters in rows denotes significant statistical differences at p < 0.05 (one-tailed *t*-test). Lowercase letters refer to CTRL-HPU 1 comparison; Capital letters refers to CTRL-HPU 2 comparison.

## 4 Conclusions

The research carried out has shown that ultrasounds can be useful for the olive paste conditioning, giving positive contribution to the global quantitative efficiency of the extraction plant. This is due to the positive contribution of sonication towards the release of oil from cell vacuoles and the positive action of ultrasound on lowering the viscosity of the pastes.

The ultrasound treatment before and after the malaxation phase did not alter the legal quality parameters of virgin olive oil. On the contrary, the quality level of the food product, strictly connected to the health and sensory properties of hydrophilic phenols, showed a significant improvement, with higher increases of phenolic content when the cavitation phenomena were activated on the crashed olive paste compared with the treatment at the end of malaxation step. For that concern the volatile fraction, the ultrasound machine determined an overall slight reduction of total content of the main volatile compounds involved in the VOO flavour. However, the increase of the only concentration of C_5_ and C_6_ saturated and unsaturated aldehydes in the HPU1 suggests the need of further investigations on the effects of HTU treatment on the LOX activity to understand the relation with different cultivar at different maturity indices and the real impact on the main sensory notes of VOO.

## CRediT authorship contribution statement

**Antonia Tamborrino:** Conceptualization, Writing - original draft. **Agnese Taticchi:** Methodology, Investigation, Writing - review & editing. **Roberto Romaniello:** Investigation, Validation, Formal analysis, Writing - review & editing. **Claudio Perone:** Investigation, Software, Writing - original draft. **Sonia Esposto:** Methodology, Investigation, Writing - original draft. **Alessandro Leone:** Conceptualization, Investigation, Supervision, Writing - original draft. **Maurizio Servili:** Conceptualization, Supervision, Writing - review & editing.

## Declaration of Competing Interest

The authors declare that they have no known competing financial interests or personal relationships that could have appeared to influence the work reported in this paper.
